# E-DNA IM or ID delivery prime enhances antibody and T cell responses following recombinant gp120 env boost

**DOI:** 10.1186/1742-4690-9-S2-P364

**Published:** 2012-09-13

**Authors:** NA Hutnick, M Karuppiah, J Pollara, J Yan, DJ Myles, K Broderick, M Morrow, N Sardasai, D Montefiori, S Barnett, G Ferrari, DB Weiner

**Affiliations:** 1 Duke University, Durham, NC, USA; 2 Inovio Pharmaceuticals, Blue Bell, PA, USA; 3 Novartis, Cambridge, MA, USA; 4 University of Pennsylvania, Philadelphia, PA, USA

## Background

The results of the RV144 trial support the ability of vaccine induced Antibodies to protect from HIV acquisition. The STEP trial also appears to have impacted viral load in individuals who produced strong T cell immunity. The HVTN has recently reported that our enhanced DNA (Pennvax) can drive T cell immunity as robust as Adenoviral and Pox platforms in an accelerated format in humans. We have now focused on further driving T and B cell immunity by utilizing prime boost

## Methods

Groups of rabbits or Indian Rhesus macaques were vaccinated with enhanced DNA vaccine plasmids encoding pGAG, pPOL + combinations of 3-5 consensus or COT designed envelopes that were either gp140 or gp160 constructs. Both IM as well as a novel minimally invasive skin delivery EP arrays (MID) were studied. DNA vaccinated animals were boosted two months following the final DNA immunization with SF162 gp120 in MF59.

## Results

In rabbits, nAb titers against a broad panel of Tier 1 viruses was observed by the combination immunization approaches. Titers in the hundreds were induced by DNA and these titers were enhanced almost a log by a protein boost. Similar data was also observed when these studies were extended to the non-human primate model. Importantly, DNA-prime protein-boost had detectable ADCC activity as measured by the GTL and luciferase assay. A bias for improved antibody responses driven by the MID delivery array is also observed.

## Conclusion

Building on the success observed in HVTN 080 we report that further enhancement of DNA constructs in a prime boost setting (E-DNA prime + protein Boost) is capable of eliciting high binding, broad antibody responses and neutralization titers against a panel of tier 1 viruses. Combining improved E-DNA with other strategies shown to enhance antibody responses such new gene adjuvants are being investigated.

